# Effect of Particle Orientation during Thermal Processing of Canned Peach Halves: A CFD Simulation

**DOI:** 10.3390/foods3020304

**Published:** 2014-05-08

**Authors:** Adreas Dimou, Nikolaos G. Stoforos, Stavros Yanniotis

**Affiliations:** Department of Food Science and Human Nutrition, Agricultural University of Athens, Athens 11855, Greece; E-Mails: dimouadreas@gmail.com (A.D.); yanniotis@aua.gr (S.Y.)

**Keywords:** Computational Fluid Dynamics, natural convection, thermal processing, liquid/particulate, peaches, particle orientation, canning, modeling

## Abstract

The objective of this work was to apply Computational Fluid Dynamics (CFD) to study the effect of particle orientation on fluid flow, temperature evolution, as well as microbial destruction, during thermal processing of still cans filled with peach halves in sugar syrup. A still metal can with four peach halves in 20% sugar syrup was heated at 100 °C for 20 min and thereafter cooled at 20 °C. Infinite heat transfer coefficient between heating medium and external can wall was considered. Peach halves were orderly placed inside the can with the empty space originally occupied by the kernel facing, in all peaches, either towards the top or the bottom of the can. In a third situation, the can was placed horizontally. Simulations revealed differences on particle temperature profiles, as well as process *F* values and critical point location, based on their orientation. At their critical points, peach halves with the kernel space facing towards the top of the can heated considerably slower and cooled faster than the peaches having their kernel space facing towards the bottom of the can. The horizontal can case exhibited intermediate cooling but the fastest heating rates and the highest *F* process values among the three cases examined. The results of this study could be used in designing of thermal processes with optimal product quality.

## 1. Introduction

Thermal processing is a widely used and extensively studied method for food preservation. Undesirable quality degradation that inevitably accompanies the targeted destruction of pathogens and spoilage agents during thermal processing of foods, calls for optimum and accurate design of a thermal process. The scientific principles for designing safe thermal processes pioneered by Ball and his colleagues [1,2] at the beginning of the previous century. These principles formed also the basis for quality retention calculations during a thermal process, calculations led by Stumbo [3,4], initiated a number of new thermal process calculation methodologies [5], among which we should mention the one presented by Hayakawa [6], and served as guide for analyzing novel preservation processes as, for example, with the case of high hydrostatic pressure processing of foods [7].

Modeling and simulation of food processes is the core of process optimization. Computational Fluid Dynamics (CFD) has been efficiently used for simulation of food processes [8,9]. A number of CFD studies refer to thermal processing of liquid/particulate systems. The majority of these investigations were focused on the analysis of fluid motion and temperature evolution. Examples include thermal processing studies on pineapple slices in juice [10], solid particles in water [11], peas in water [12] and asparagus in brine [13]. The use of CFD for microbial destruction calculations during thermal processing (for table olives in brine) has been also reported [14].

A number of processing schedules, depending on can size, processing method (stationary *v*s. rotary systems) method of vacuum formation (thermal *vs*. mechanical) *etc.*, have been reported in the literature for peach canning [15]. Thus, for example, processing for 20 to 25 min is recommended for syrup packed peach halves in 401 × 411 cans still processed in boiling water, when exhausted to a temperature of 71.1 °C (160 °F). Cans using mechanical means to obtain vacuum require 5 to 10 min longer heating time due to their lower initial temperature. Alternatively, achieving a minimum temperature of 87.8 °C (190 °F) at the end of the heating cycle, at the critical point of the product, before air cooling, or of 90.6 °C (195 °F) before water cooling, is proposed [15]. Knowledge of heat transfer characteristics, as well as of the location of the critical point, that is, the point that receives the least effect, as far as microbial destruction is concerned, of the heat treatment, is essential in designing thermal processes for such products.

Particle orientation can significantly influence liquid motion and heat transfer rates in liquid/particulate systems during natural convection heating. The objective of this work was to apply Computational Fluid Dynamics in studying the flow field and the temperature profile, as well as microbial inactivation, in thermally processed still cans filled with peach halves in sugar syrup.

## 2. Experimental Section

**Figure 1:**
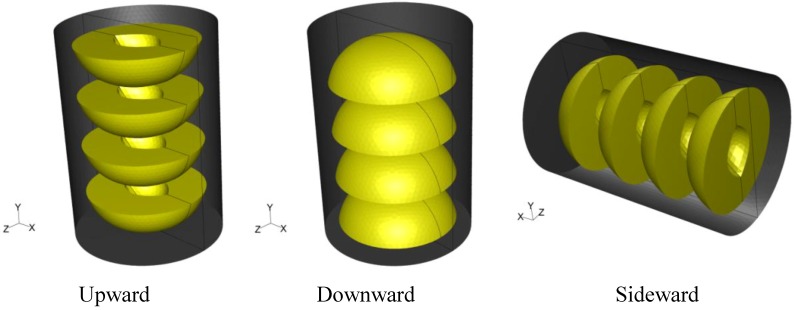
Three dimensional arrangement and orientation of cans and peach halves in the cans.

A tin can with dimensions of 7.5 cm in diameter and 10.5 cm in height filled with syrup (20% sugar) and four peach halves was used in all simulations. Peach halves were orderly placed inside the can. In two cases, the can was placed vertically having the peach halves with the empty space originally occupied by the kernel facing, in all peaches, either towards the top (termed hereafter “upward”) or the bottom of the can (termed hereafter “downward”). In a third case, the vertical can having the peach halves with the empty space originally occupied by the kernel facing towards the bottom of the container was placed horizontally (turned 90 degrees clockwise, termed hereafter “sideward”). The system was designed with mid-plane symmetry. The three dimensional arrangement and orientation of cans and peach halves in the cans are shown in Figure 1. Note that the *x*–*z* plane at the presentation of the horizontal can on Figure 1 is reversed, in order for the geometry of the empty space originally occupied by the kernel to be visible.

The can with its contents were initially at rest and at uniform temperature (20 °C), heated in a medium at constant temperature (100 °C) for 20 min and cooled at constant medium temperature of 20 °C. The internal heat transfer coefficient between the filling liquid (syrup) and internal can wall (based on natural convection heating of the syrup) is expected to create the main resistance to heat transfer. Thus, infinite heat transfer coefficient between heating or cooling medium and external can wall was considered. Negligible resistance to heat transfer of the metal wall, and no slip at the container’s wall, was further assumed. Only heat transfer from the can to the syrup and from the syrup to peaches was considered.

For the simulation of the fluid field and the temperature evolution in the product (peaches and syrup), the partial differential equations of mass, momentum and energy conservation [16] were solved using the software FLUENT 6.3.26 (Fluent Ansys Inc., Canonsburg, PA, USA, 2006) with 3D, double precision, pressure-based, laminar flow (due to the Rayleigh number being less than 10^8^ during the heating and cooling cycle). Mid-plane symmetry (as depicted on Figure 1) was assumed. Axi-symmetric simulation could not be applied for sideward case. The time step used was equal to 0.5 s, the Courant number was equal to 0.5, the algorithm of pressure-velocity coupling was “Coupled” and the model used for the discretization of pressure was “PRESTO!”. For the discretization of momentum and energy equations, the model “First Order Upwind” was selected. Preliminary trials showed no differences in temperature predictions between first- and second- order upwind models. In FLUENT nomenclature, the internal surface of the can, as well as the external surface of the peach halves, was defined as wall. The volume of the peach halves was considered as solid. The volume between the peach halves and the can, occupied by the syrup, was considered as fluid. The volumes of the syrup and the peach halves were designed in Gambit^®^ 2.3.16.

Peach shapes were designed to resemble, as close as possible, the real product geometry. Thus peach halves were designed as semi oblate spheroids with 6.0 cm in large diameter and 4.0 cm in small diameter. The kernels were designed as semi ellipsoids with 2.0 cm in the middle axis (corresponding to kernel’s depth) and 3.0 cm in the largest axis and 1.5 cm in the smallest axis, resulting in uneven thickness of the peaches (1.5 cm in the *x* axis, 2.25 cm in the *z* axis, and 1 cm in the *y* axis, Figure 1).

The shape of the grid was “Tet/Hybrid” for both peach halves and syrup volumes with the option of 18% “Shortest edge” of the software. The latter means that a value equal to the 18% of the size of the smallest edge existing in the entire geometry is used to calculate the total number of intervals at all edges. Gambit^®^ calculates the number of intervals on any edge by dividing the size of the particular edge by this value. For the half can volume (due to the assumed symmetry only half of the can was simulated) and for the case of “upward” peach halves, 74,770 cells (for both peach halves and syrup) were used for the discretization and solution of the governing equations, while for the “downward” and the “sideward” peach halves 73,364 and 73,590 cells, respectively were employed.

The thermo-physical properties of 20% (w/v) sugar aqueous solution obtained from literature were used for the syrup properties. In the temperature range of 20 °C to 130 °C, values for density (*ρ*), viscosity (*μ*) and specific heat (*C_p_*) were obtained through on-line calculations [17], while thermal conductivity (*k*) values were read from [18]. Based on those values, each property was expressed as a function of temperature (*T*, in °C) through a second or third order polynomial equation (derived in Microsoft^®^ Office Excel) as shown on Table 1. Peach properties from literature [19] were assumed constant throughout the process and equal to the mean values presented on Table 1.

**Table 1:** Physical and thermal properties of liquid and solids used in simulations.

	Syrup (20% w/v sugar)	Peach
*ρ* (kg/m^3^)	1.0868 × 10^3^ − 2.4377 × 10^−1^ × *T* − 2.4201 × 10^3^× *T*^2^ (*R*^2^ = 0.9985)	1022 ± 3
*μ* (Pa·s)	3.3817 × 10^−3^ − 8.2745 × 10^−5^ × *T* + 8.3164 × 10^−7^ × *T*^2^ − 2.8823 × 10^−9^× *T*^3^ (*R*^2^ = 0.9955)	-
*C_p_* (J/kg·K)	3.6839 × 10^3^ + 1.4510 × 10^0^ × *T* + 3.4965 × 10^−4^ × *T*^2^ (*R*^2^ = 0.9980)	3992 ± 7
*k* (W/m·K)	5.0486 ×10^−1^ + 1.5929 × 10^−3^ × *T* − 5.0000 × 10^−6^ × *T*^2^ (*R*^2^ = 0.9999)	0.58 ± 0.09

Based on the classical thermobacteriological approach [20], the *F* value of the process was calculated through Equation (1):

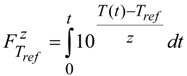
(1)


where the 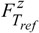 value is defined as the equivalent processing time of a hypothetical thermal process at a constant reference temperature, *T_ref_*, that produces the same effect (in terms of microbial, or of any other heat sensitive substance destruction) as the actual thermal process, the *z* value is the temperature difference required to achieve a tenfold change of the decimal reduction time of the heat sensitive substance, and *T* being the temperature (at a particular point in the product) and *t* the processing time.

Calculations were based on a *z* value of 11.5 °C (indicative to *Clostridium butiricum*, [21]) and a reference temperature of 90 °C, appropriate for thermal treatment of acid foods. A User Defined Function (UDF) was written and imported to FLUENT in order to calculate *F* values at every point inside the container where temperature values were calculated. For the completion of the calculation procedure described so far, computational time was about 3 days using a personal computer with Intel^®^ Core TM 2, CPU 660 @ 2.40 GHz and 2048 MB RAM.

## 3. Results and Discussion

**Figure 2:**
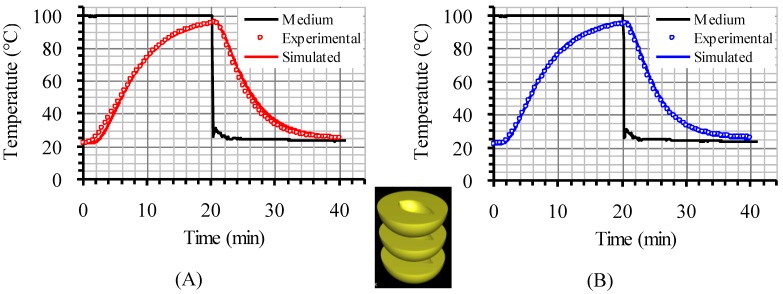
Experimental and simulated temperature data at the interior of the central (**A**) and the bottom peach half (**B**), in reference to the inserted picture, in a can containing three peach halves in 20% sugar syrup.

The model used in the present investigation was validated for various cases, such as asparagus (two experimental set-ups in triplicate), table olives (duplicate experiments) and peach halves (single run) in liquid, processed in still cans, in earlier works [13,14,22]. In brief, and for the case of peach halves, the temperature inside (at predescribed locations) the bottom and the central peach halves, in a can containing three peach halves, closely packed, in 20% sugar syrup, heated in boiling water, was measured with type K thermocouples and compared (successfully) to model predictions (Figure 2). Validation of model’s predictions with experimental data for the setup with four peach halves analyzed in the present study was not performed and this could be considered as limitation of the present work.

### 3.1. Velocity Profile

**Figure 3:**
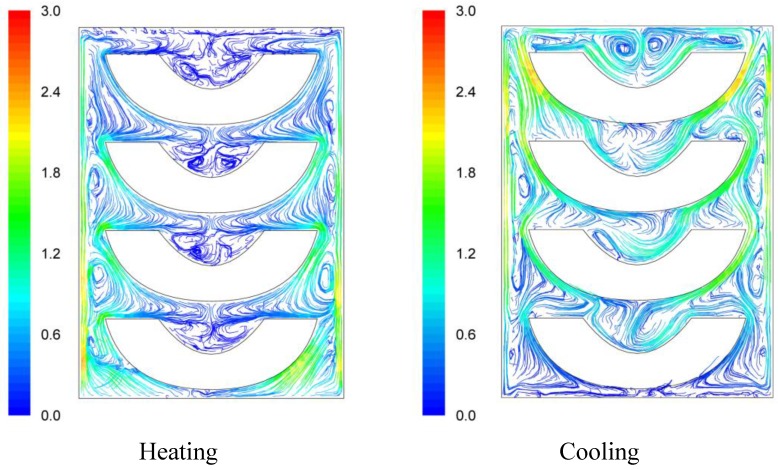
Velocity pathlines after 30 s of heating (left) and after 30 s of cooling (right) for the “upward” case (velocity in cm/s).

**Figure 4:**
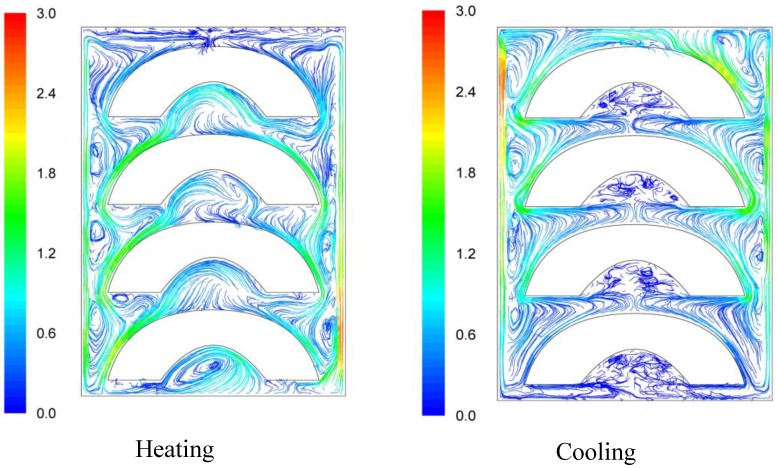
Velocity pathlines after 30 s of heating (left) and after 30 s of cooling (right) for the “downward” case (velocity in cm/s).

**Figure 5:**
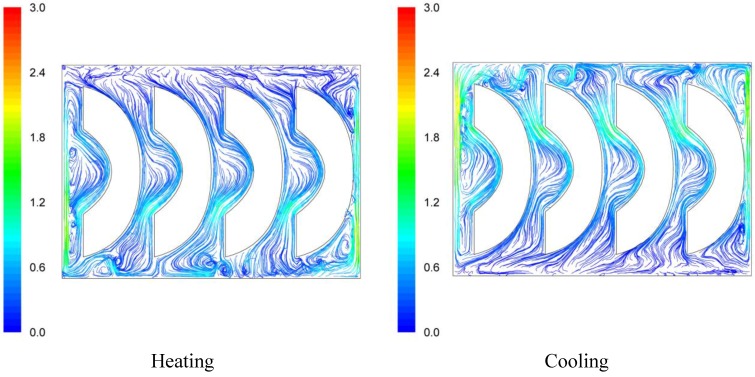
Velocity pathlines after 30 s of heating (left) and after 30 s of cooling (right) for the “sideward” case (velocity in cm/s).

Velocity pathways, indicative to fluid motion due to buoyancy, are presented on Figure 3, Figure 4 and Figure 5, 30 s after the beginning of heating and 30 s after the beginning of cooling, for the cases studied. In all cases, during heating, hot syrup close to can vertical walls moves towards the top of the container forcing the syrup on the top of the container to move downwards at the interior of the container between the solid peaches. Thirty seconds after the onset of heating, indicative velocities (maximum calculated values) were 2.568 cm/s, 2.760 cm/s and 2.147 cm/s for the “upward”, “downward” and “sideward” cases, respectively. Hot syrup heats the cold peach halves when in contact. The whole process is repeated until no temperature differences within the syrup exist.

During cooling the direction of fluid motion is reversed, for the syrup being moving downwards near the wall because of the fluid’s cooling and upwards in the interior. Thirty seconds after the onset of cooling, maximum calculated velocities were 2.838 cm/s, 2.709 cm/s and 2.301 cm/s for the “upward”, “downward” and “sideward” cases, respectively.

### 3.2. Temperature Profile

**Figure 6:**
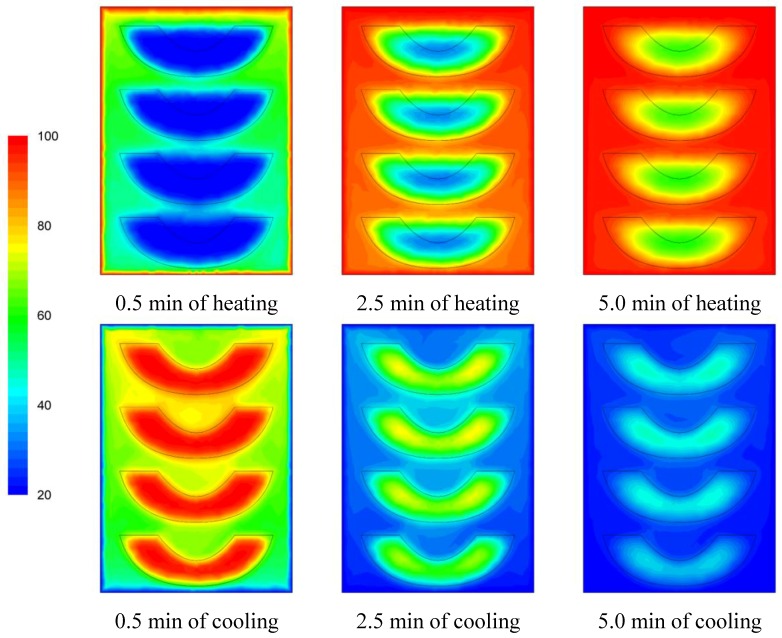
Temperature (°C) contours for peach halves in syrup at different heating and cooling times during heating (at 100 °C) and cooling (at 20 °C) for the “upward” case.

**Figure 7:**
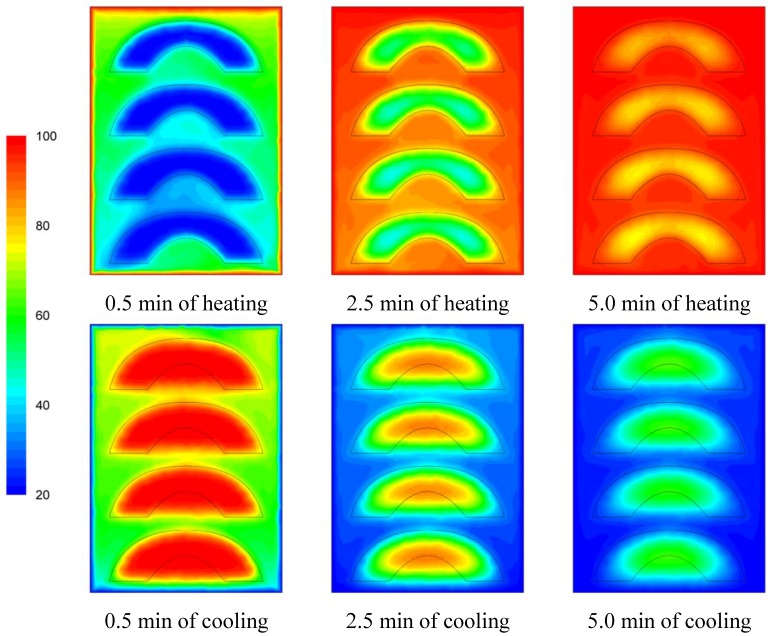
Temperature (°C) contours for peach halves in syrup at different heating and cooling times during heating (at 100 °C) and cooling (at 20 °C) for the “downward” case.

**Figure 8:**
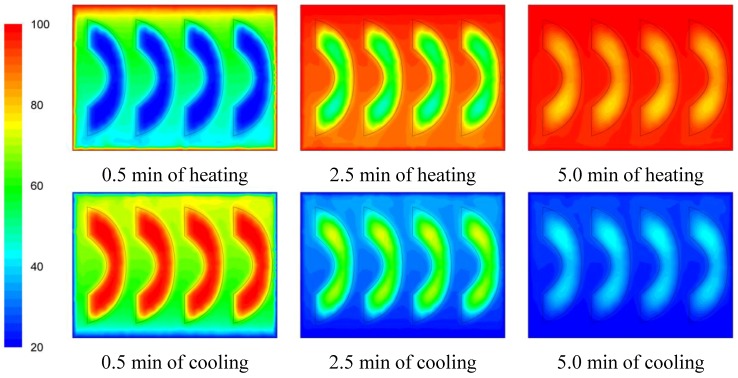
Temperature (°C) contours for peach halves in syrup at different heating and cooling times during heating (at 100 °C) and cooling (at 20 °C) for the “sideward” case.

The temperature evolution during heating and cooling of the product is indicatively shown on Figures 6, Figure 7 and Figure 8 at selected processing times, for the three cases studied. Heating and cooling rates are a direct consequence of the syrup motion which, by its turn, is greatly affected by the orientation of the can (vertical *vs*. horizontal) and the arrangement of the peach halves within the can (having the empty space originally occupied by the kernel facing either towards the top or the bottom of the can). The contents of the horizontal can (“sideward” case) were heated at comparable rates or slightly faster compared to the “downward” case of the vertical container (as can be seen on Figure 7 and Figure 8 after 5 min of heating) with the latter (the “downward” case) heating faster compared to the “upward” case (as can be seen on Figure 6 and Figure 7 after 5 min of heating). During cooling, slower rates were observed for the “downward” case, followed by the “sideward” and “upward” cases which cool in comparable rates (see Figures 6, Figure 7 and Figure 8 after 5 min of cooling).

Differences between the “upward” and the “downward” cases in heating and cooling rates are related to the corresponding differences in fluid flow. During heating, fluid velocities at the cavity of the peach halves for the “upward” case are low (Figure 3) resulting in a rather stagnant, slow heated pool of liquid. For the “downward” case, natural convection currents at the peach cavities (Figure 4) result to higher heating rates compared to the “upward” case. During cooling, descending natural convection currents at the peach cavities for the “upward” case (Figure 3) result to higher cooling rates compared to the “downward” case, where hot fluid is trapped into the peach cavity (Figure 4). Thus, the “upward” case heats faster and cools slower than the “downward” case, resulting to more intense thermal treatment. The unrestricted motion of the fluid on both sides of the peach halves for the “sideward” case (Figure 5) results in higher heat transfer rates from fluid to the particles during both heating and cooling compared to the other two cases.

Based on temperature calculations at each grid point, the slowest heating zone during the heating cycle and the slowest cooling zone during the cooling cycle of the thermal process were identified. Thus, for example, the exact rectangular coordinates (*x*, *y*, *z*) in mm, for the slowest heating point for the cases studied were: (0, −40.0, 0) for the “upward”, (−7.6, −35.8, −7.9) for the “downward” and (−35.6, −13.5, 0) for the “sideward” facing peach halves, respectively. Note that the origin, *x* = 0, *y* = 0 and *z* = 0 refer to the geometric center of the can, while in reference to Figures 6, Figure 7 and Figure 8, positive *x* is measured as we move from the center to the right, positive *y* is measured as we move from the center to the top and positive *z* is measured as we move from the center outwards of the paper (see Figure 1).

### 3.3. *F* Value Distribution

**Figure 9:**
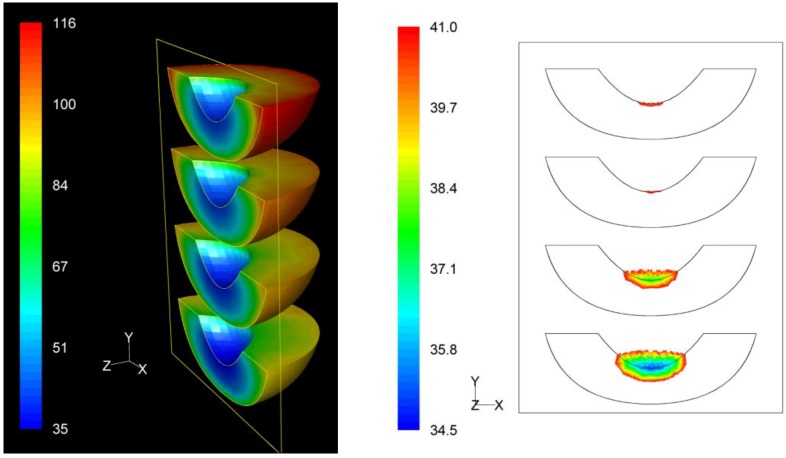
*F* value (in min) distribution within the peach halves (left) and location of the critical point (right) at the end of the thermal process for the “upward” case.

**Figure 10:**
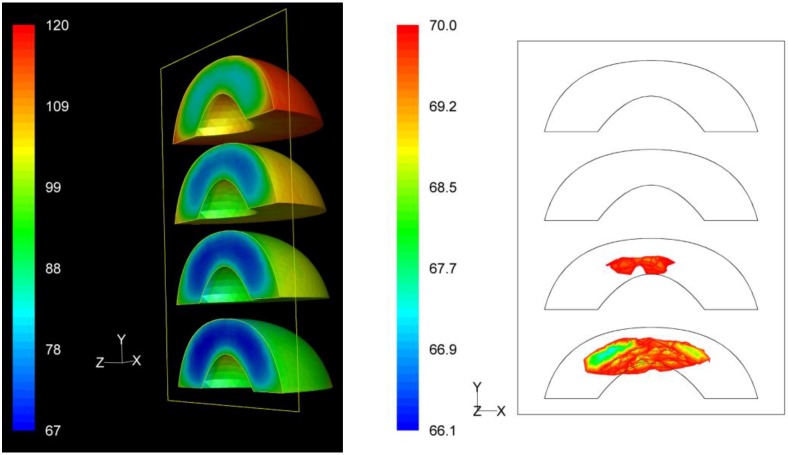
*F* value (in min) distribution within the peach halves (left) and location of the critical point (right) at the end of the thermal process for the “downward” case.

**Figure 11:**
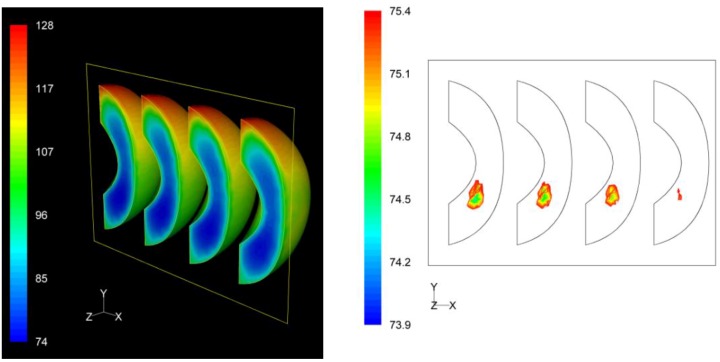
*F* value (in min) distribution within the peach halves (left) and location of the critical point (right) at the end of the thermal process for the “sideward” case.

Based on process *F* value calculations at each nodal point, the distribution of the *F* values within the product throughout the whole process could be assessed. The distribution of the *F* values as well as the precise location of the critical zone, that is the region in the product receiving the least effects of the process, in terms of microbial destruction, are depicted on Figures 9, Figure 10 and Figure 11 for the three peach arrangements examined. The process *F* values ranges for the peach halves for the “upward” “downward” and “sideward” cases, were 34.9 min to 116.4 min, 66.1 min to 119.9 min, and 73.9 min to 128.1 min, respectively. As can be seen from the left pictures on Figures 9, Figure 10 and Figure 11, the highest *F* values were calculated at the surface of the peaches located at the top part of the can.

If we focus at the interior of the peach halves, the lowest *F* values calculated for the four peach halves of the “upward” case were 34.9 min, 37.8 min, 40.9 min and 40.6 min as we move from the bottom towards the top of the can (right picture on Figure 9). Note that these *F* values are rather narrowly distributed. The critical point was located at the peach half at the bottom of the can, with exact coordinates (*x*, *y*, *z*) in mm being (0, −40.0, 0). Note that for this case the critical point coincides with the slowest heating point discussed earlier.

For the “downward” case, the lowest *F* values calculated at the interior of the four peach halves peach halves were 66.1 min, 69.2 min, 74.2 min and 81.3 min as we move from the bottom towards the top of the can (Figure 10). Note that these *F* values are rather dispersed. The critical point was located at the peach half at the bottom of the can, with exact coordinates (*x*, *y*, *z*) in mm being (−10.2, −35.9, −9.1). Note that for this case the location of the critical point differs slightly from the location of slowest heating point (−7.6, −35.8, −7.9) presented earlier.

Finally, for the “sideward” case, the lowest F values calculated at the interior of the four peach halves were 73.9 min, 74.6 min, 74.8 min and 75.2 min as we move from left to right on the right picture on Figure 11. Note that these F values are practically identical. The critical point was located at the first peach half from the left, with exact coordinates (*x*, *y*, *z*) in mm being (−35.6, −13.5, 0). As for the “upward” case, for this case also the critical point coincides with the slowest heating point.

For the simulations presented, heating for 20 min at 100 °C (followed by cooling at 20 °C) was used. This process schedule resulted to high *F* process values for all cases. A realistic target *F* value can be set by requiring a six-log reduction of *Clostridium* *butyricum* population, characterized by a decimal reduction time at 90 °C equal to 1.1 min [23]. This leads to a target 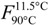 value of 6.6 min. Given the existence of a distribution of *F* values, if one had to calculate heating times for a particular microbial destruction, then appropriate calculations should be performed at the critical point for each case in order to avoid underprocessing.

**Figure 12:**
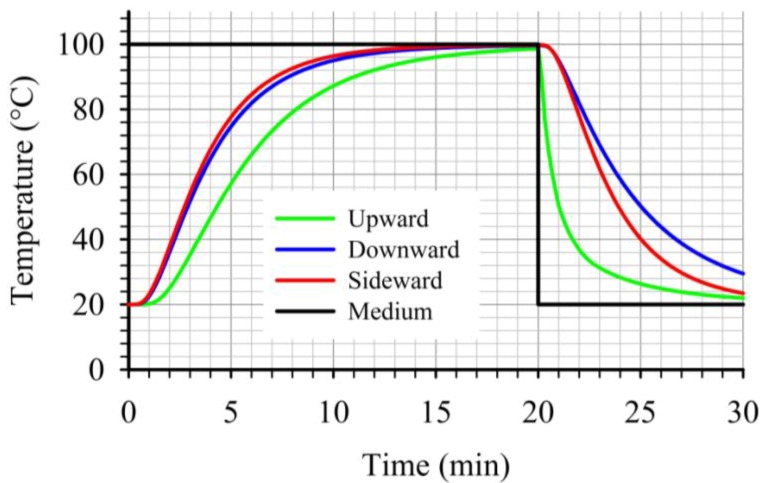
Comparison of temperature profiles at the critical point during thermal processing of peach halves of different orientations in syrup (20% sugar) in stationary metal cans.

At the critical point, for the three cases studied, the “upward” case resulted to lower heat transfer rates during heating as illustrated on Figure 12. Furthermore, during cooling, the temperature at the critical point for the “upward” case dropped noticeably faster compared to the other two cases (Figure 12). This led to the lowest *F* values, at the critical point, within the three cases studied. Thus, for a given target *F* value, the “upward” case will require longer heating times compared to the other two cases studied. Comparing the temperature profiles, at the critical points, for the other two cases, one can note the slightly faster heating and cooling for the “sideward” case compared to the “downward” case. Due to the fact that at the beginning of the cooling the temperatures at the critical points for the two cases were comparable, the faster heating rates associated with the “sideward” case caused the accumulation of higher *F* values for this case.

## 4. Conclusions

The CFD simulations of thermal processing of four peach halves in 20% w/v sugar syrup in stationary cans gave insights as far as fluid motion, temperature evolution, microbial *F* value distribution and location of the critical point. For the same processing time, the “sideward” case recorded the highest and the most uniform, within the peach halves, *F* process values. Comparing the two cases of the vertical can with the peach halves placed with the empty space originally occupied by the kernel facing towards the top (the “upward” case) or the bottom of the can (the “downward” case) slower heating but faster cooling rates were calculated for the “upward” case, fact that led to lower *F* process values for the “upward” case. The *F* value at the critical points within each peach half for the “upward” case was more uniform compared to the “downward” case.

For the “upward” case, the critical point was located at the upper part (where the kernel was located) of the peach half at the bottom of the can. For the “downward” case, the critical point was located at the interior of the peach half at the bottom of the can. Finally, for the “sideward” case the critical point was at the lower portion (at about 1/3 of the peach diameter) of the first peach half from the left (that is, the peach half having the empty space originally occupied by the kernel facing towards from the can bottom). A number of parameters, such as, heating time and temperature, target *F* value and microbial *z* value, size and number of peach halves, particle arrangement—including random placement, can be further investigated based on the presented principles and approach.
